# Forebrain delta opioid receptors regulate the response of delta agonist in models of migraine and opioid-induced hyperalgesia

**DOI:** 10.1038/s41598-020-74605-9

**Published:** 2020-10-19

**Authors:** Isaac J. Dripps, Zachariah Bertels, Laura S. Moye, Alycia F. Tipton, Kendra Siegersma, Serapio M. Baca, Brigitte L. Kieffer, Amynah A. Pradhan

**Affiliations:** 1 Department of Psychiatry, University of Illinois at Chicago, 1601 W. Taylor Street (MC 912), Chicago, IL 60612 USA; 2 Department of Pharmaceutical Sciences, University of Colorado Anschutz Medical Campus, Aurora, USA; 3 Department of Neurology, University of Colorado Anschutz Medical Campus, Aurora, USA; 4 Department of Psychiatry, Douglas Mental Health University Institute, McGill University, Montreal, Canada

**Keywords:** Chronic pain, Chronic pain

## Abstract

Delta opioid receptor (DOR) agonists have been identified as a promising novel therapy for headache disorders. DORs are broadly expressed in several peripheral and central regions important for pain processing and mood regulation; and it is unclear which receptors regulate headache associated symptoms. In a model of chronic migraine-associated pain using the human migraine trigger, nitroglycerin, we observed increased expression of DOR in cortex, hippocampus, and striatum; suggesting a role for these forebrain regions in the regulation of migraine. To test this hypothesis, we used conditional knockout mice with DORs deleted from forebrain GABAergic neurons (Dlx-DOR), and investigated the outcome of this knockout on the effectiveness of the DOR agonist SNC80 in multiple headache models. In DOR loxP controls SNC80 blocked the development of acute and chronic cephalic allodynia in the chronic nitroglycerin model, an effect that was lost in Dlx-DOR mice. In addition, the anti-allodynic effects of SNC80 were lost in a model of opioid induced hyperalgesia/medication overuse headache in Dlx-DOR conditional knockouts. In a model reflecting negative affect associated with migraine, SNC80 was only effective in loxP controls and not Dlx-DOR mice. Similarly, SNC80 was ineffective in the cortical spreading depression model of migraine aura in conditional knockout mice. Taken together, these data indicate that forebrain DORs are necessary for the action of DOR agonists in relieving headache-related symptoms and suggest that forebrain regions may play an important role in migraine modulation.

## Introduction

According to the most recent Global Burden of Disease Study, headache was ranked as third most prevalent and a common type of headache, migraine, was ranked as sixth most prevalent worldwide^1^. Although opioids are not recommended for migraine, in the US opioid analgesics that primarily target the µ opioid receptor are still prescribed for this disorder. A recent large scale epidemiological study of chronic migraine patients found that 36.3% of respondents used or kept on hand opioid medications for treatment of their headaches^2^. However, µ agonists have poor efficacy for migraine, high abuse liability, and prolonged use can exacerbate pain symptoms leading to conditions such as medication overuse headache (MOH) or opioid-induced hyperalgesia (OIH)^3–6^.

The δ opioid receptor shows promise as a novel target for migraine treatment^7^. In mice, δ agonists have been shown to inhibit mechanical^8^ and thermal^8,9^ allodynia produced by nitroglycerin (NTG), a known human migraine trigger^10,11^. The δ agonist SNC80 has also been shown to inhibit NTG-induced conditioned place aversion, a model of migraine-associated negative affect, and cortical spreading depression (CSD), a well-characterized model of migraine with aura (Pradhan et al.^8^). Recent evidence indicates that δ agonists may be effective in headache disorders more broadly. SNC80 was found to inhibit allodynia associated with post-traumatic headache, MOH induced by overuse of sumatriptan, and OIH^12,13^.

To further develop δ agonists for the treatment of headache disorders, it is important to understand where and how δ receptors inhibit distinct but related migraine symptoms. Along with their expression in pain processing areas, δ receptors are highly expressed in several brain regions important for processing the emotional content of pain and mood regulation including the cortex, hippocampus, and striatum^14–16^. The relative contributions of δ receptors to the regulation of pain signaling has been evaluated in several models but has been mainly restricted to examinations of peripheral δ receptors. In a conditional knockout mouse with δ receptors deleted from peripheral voltage-gated sodium channel Na_v_1.8-expressing neurons (Na_v_1.8-DOR), δ agonist-induced anti-hyperalgesic effects were abolished in the Complete Freund’s Adjuvant (CFA) and the sciatic nerve ligation models^17,18^. These data indicate that peripheral δ receptors are critical for regulating peripheral inflammatory and neuropathic pain, respectively. Conversely, in a migraine model the peripherally restricted opioid antagonist N-methylnaltrexone failed to block SNC80-induced inhibition of NTG-evoked thermal hyperalgesia, suggesting a role for central δ receptors in migraine-associated pain^9^.

In order to clarify the role of central δ receptors in migraine, we first examined the effect of chronic migraine-associated pain on δ receptor expression in key forebrain regions. We then used Dlx-DOR conditional knockout mice in which δ receptors are deleted specifically from forebrain GABAergic neurons^19,20^. These mice were used to examine the effect of Dlx5/6-DOR conditional knockout on the ability of the δ-selective agonist SNC80 to inhibit NTG-induced allodynia and negative affect, as well as OIH, and CSD.

## Results

### Effect of NTG on DOR-eGFP expression

**Figure 1:**
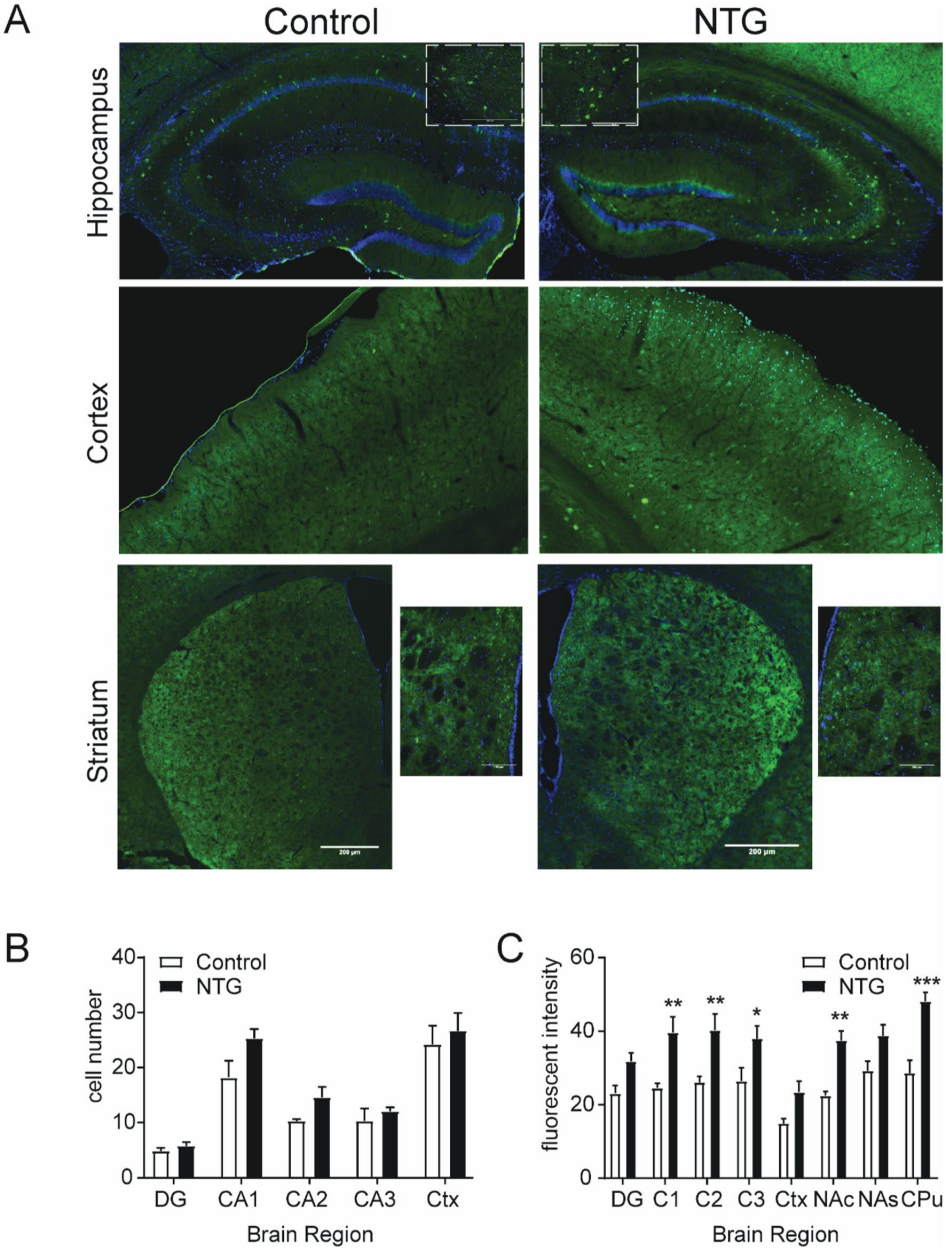
Chronic intermittent NTG increases DOR-eGFP expression. Mice were treated every other day for 9 days with vehicle of NTG (10 mg kg^−1^ IP) and tissue was collected on day 10. (**A**) Representative ×10 images of saline and NTG treated brain regions. Hippocampus, inset—×20 zoom of representative neurons from the CA3 region of the hippocampus. Somatosensory Cortex, Striatum, inset—×20 zoom of representative image. (**B**) Comparison of number of DOR-eGFP positive cells in different brain regions of saline and NTG treated mice. (**C**) Average fluorescent intensity of DOR-eGFP positive cells in different brain regions of saline and NTG treated mice. For striatal regions overall fluorescence was quantified. *DG* Dentate gyrus, *CA1* Region 1 of the hippocampus, *CA2* Region 2 of the hippocampus, *CA3* Region 3 of the hippocampus, *SC* primary somatosensory cortex, *NAC* nucleus accumbens core, *NAS* nucleus accumbens shell, *Cpu* caudate putamen *p < 0.05, **p < 0.01, ***p < 0.001 compared to saline treated group in the same region. Multiple t-test with Holm-Sidak correction. Specific p values can be found in Supplementary Table 1. n = 5/group.

δ receptors are abundantly expressed in forebrain regions such as the hippocampus, somatosensory cortex, and striatum. We used the DOR-eGFP knockin mouse to evaluate the effects of chronic-migraine associated pain on δ receptor expression in these brain regions specifically. We used the nitroglycerin (NTG) model of chronic migraine in which mice are treated every other day for 9 days with vehicle or NTG^21^. Similar to C57BL6 mice^21^, chronic NTG treatment resulted in a significant basal/chronic allodynia in DOR-eGFP mice (Supplementary Fig. 1). There was high expression of DOR-eGFP in the hippocampus, striatum, and cortex (Fig. 1A). NTG treatment did not result in a significant change in the number of DOR-eGFP positive cells in any region examined (Fig. 1B, Supplementary Table 1). However, the average fluorescent intensity of DOR-eGFP positive cells in the hippocampus and somatosensory cortex of NTG treated mice was significantly increased relative to vehicle treated controls (Fig. 1C, Supplementary Table 1). DOR-eGFP fluorescence in the nucleus accumbens (NAc) and caudate putamen (CPu) was too dense to consistently identify individual cell bodies in these regions. However, the average fluorescent intensity of these regions was significantly increased in the brains of NTG treated mice relative to saline treated controls (Fig. 1C, Supplementary Table 1). We also determined if chronic NTG treatment affected δ expression in the trigeminal nucleus caudalis (TNC) and trigeminal ganglia (TG), two regions that are important for head pain processing; and we also observed an upregulation in these regions (personal communication, publication pending). These results indicate that δ receptors in multiple regions, including the forebrain are affected by chronic migraine-associated pain.

### Confirmation of Dlx-DOR conditional knockout

**Figure 2:**
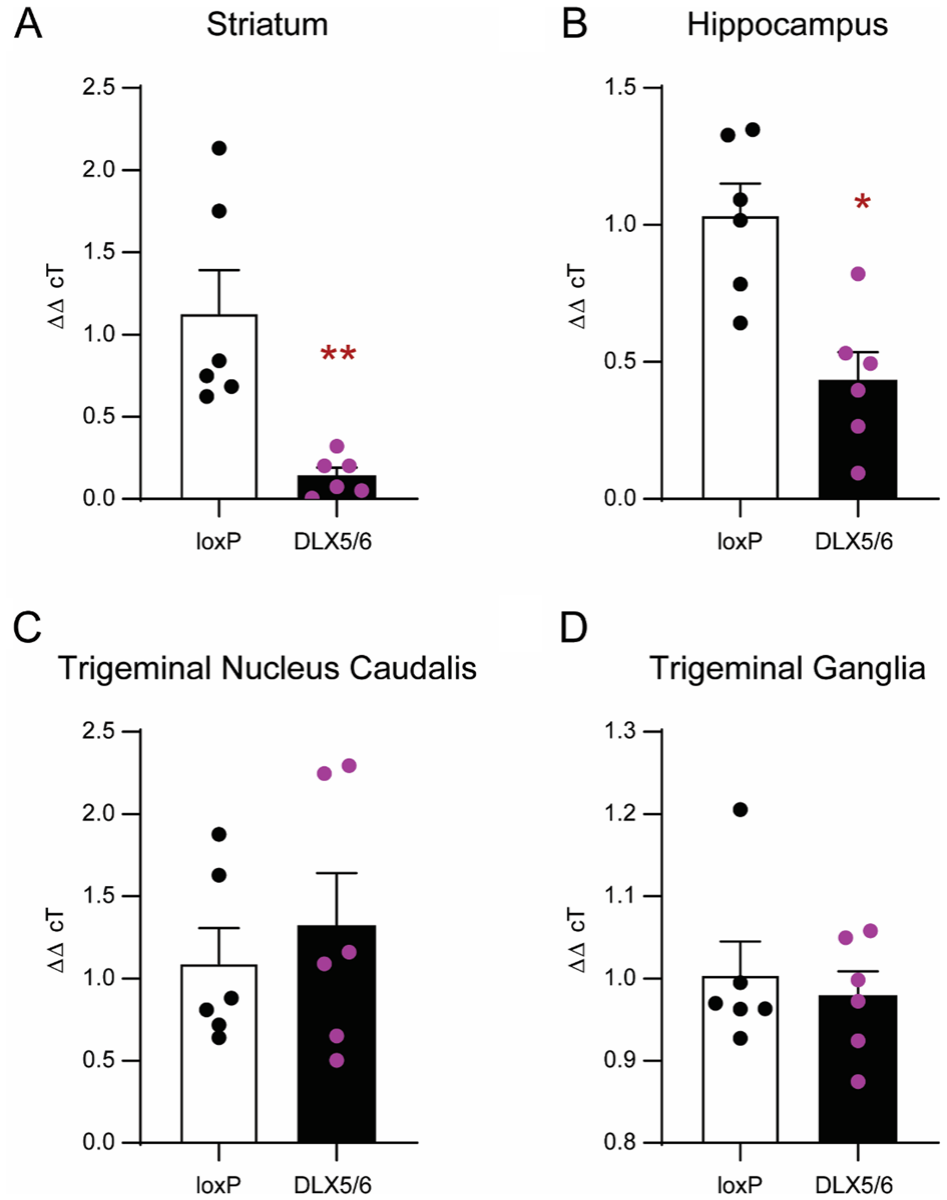
Expression of δ receptors in Dlx-DOR conditional knockouts. Quantitative RT-PCR was performed on (**A**) striatum (**B**) hippocampus (**C**) trigeminal nucleus caudalis (TNC) and (**D**) trigeminal ganglia (TG) taken from adult loxP and Dlx-DOR mice. Multiple t-tests with Holm-Sidak correction showed a significant reduction of DOR in the two forebrain regions (*p < 0.05, **p < 0.01). Specific p values can be found in Supplementary Table 1. n = 6/genotype.

The conditional knockout induced by crossing floxed mice with Dlx5/6-Cre (Dlx-DOR) mice has been previously described^19,20^. To confirm that we obtained a similar knockout, we determined gene expression of δ receptors in regions that were shown to be affected by this cross. Similar to the initial characterization of these animals, we also observed a ~ 80% and ~ 50% decrease in δ transcript levels in Dlx-DOR mice in the striatum and hippocampus, respectively (Fig. 2A,B, Supplementary Table 1). As we are investigating the role of δ receptors in headache disorders, we also examined the level of δ receptor gene expression in the TNC and TG. In this case, Dlx-DOR mice showed similar δ expression relative to loxP controls (Fig. 2C,D, Supplementary Table 1). These results confirm that the Dlx-DOR mice used in these experiments showed a conditional knockout of δ receptor in forebrain regions and maintained δ receptor expression in hindbrain and peripheral regions.

### Role of central δ receptors in NTG-induced allodynia

**Figure 3:**
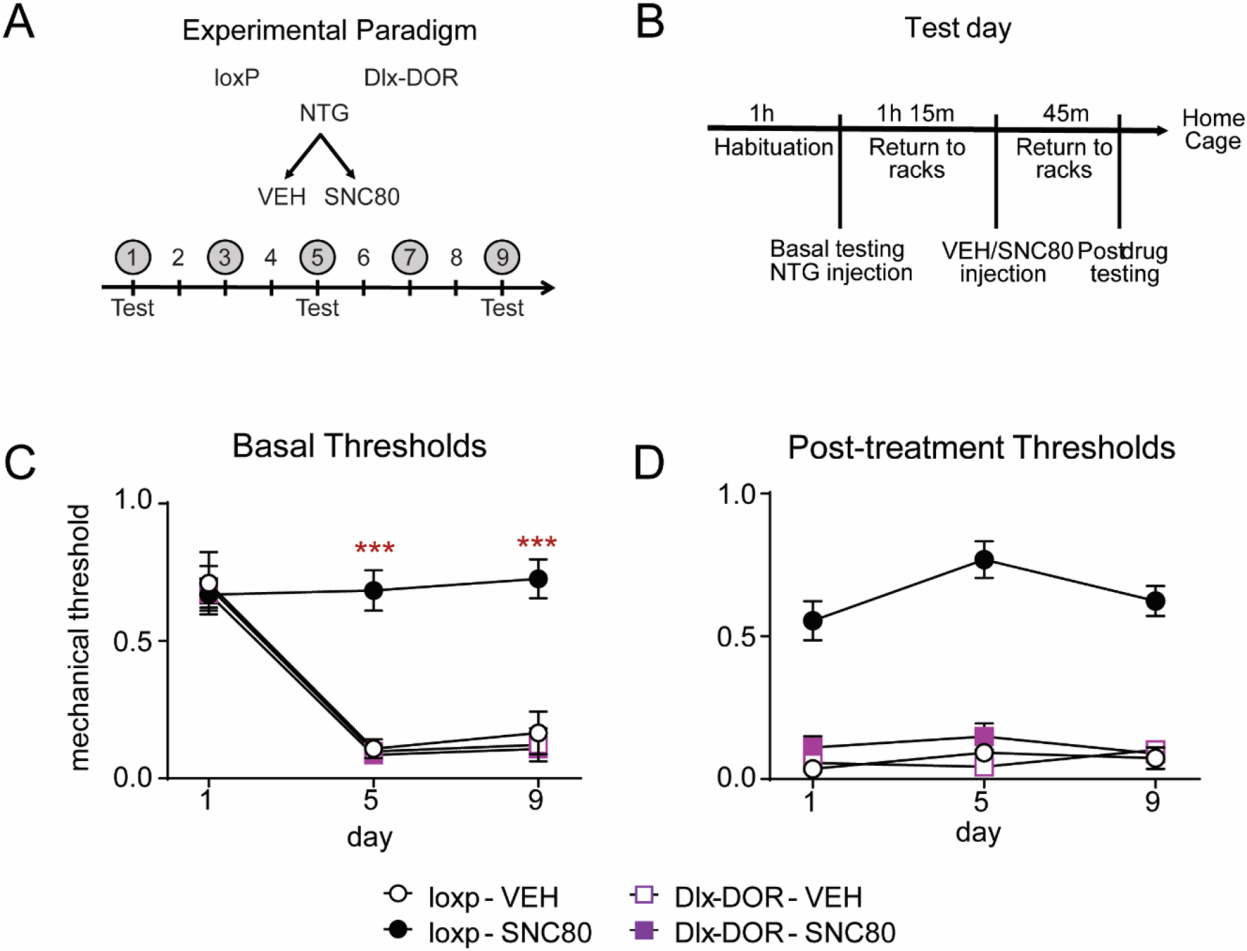
Role of central δ receptors in NTG-induced periorbital allodynia. (**A**) Mice were injected every other day for 9 days with vehicle or NTG (10 mg kg^−1^ IP) and 1 h 15 min later with vehicle or SNC80 (10 mg kg^−1^ IP). (**B**) They were tested for basal and post-treatment cephalic responses on days 1, 5, and 9. Repeated administration of NTG produced (**C**) basal and (**D**) acute periorbital allodynia in loxP and Dlx-DOR mice. (**C**) SNC80 prevented the development of basal hypersensitivity in loxP controls; an effect that was not observed in Dlx-DOR mice. Three-way repeated measures ANOVA revealed significant effects of day (F(2,2) = 62.43, p < 0.001), SNC80 dose (F(1,2) = 23.37, p < 0.001), and genotype (F(1,2) = 35.28, p < 0.001), as well as significant interaction of day X SNC80 dose (F(2,2) = 8.32, p = 0.0006), day X genotype (F(2,2) = 8.46, p = 0.0006), SNC80 dose X genotype (F(1,2) = 28.42, p < 0.001), and day X SNC80 dose X genotype (F(2,2) = 7.70, p = 0.001). (**D**) SNC80 inhibited the acute allodynic effects of NTG in loxP controls, but failed to do so in Dlx-DOR mice. Three-way repeated measures ANOVA revealed significant effects of SNC80 dose (F(1,2) = 181.4, p < 0.001), genotype (F(1,2) = 129.5, p < 0.001), and a significant SNC80 dose X genotype interaction (F(1,2) = 129.4, p < 0.001). n = 6/group for all panels. ***p < 0.001 compared to all other groups on the same day. n = 6/group.

We have previously demonstrated that the δ agonist SNC80 alleviates NTG-induced mechanical allodynia. To investigate the relative contribution of central δ receptors to this effect, we evaluated the anti-allodynic effects of SNC80 in Dlx-DOR knockout mice using the chronic intermittent NTG model of chronic migraine. On days 1, 3, 5, 7, and 9, mice were treated with either VEH or NTG (10 mg kg^−1^ IP), and then injected with VEH or SNC80 (10 mg kg^−1^ IP) 45 min later (Fig. 3A). Cephalic/periorbital allodynia was only determined on days 1, 5 and 9. On each test day both basal (prior to injections that day) and post-treatment thresholds were determined (Fig. 3B). Dlx-DOR knockout mice did not differ from loxP littermate controls in terms of naïve baseline cephalic responses (Fig. 3C, day1) or in the cephalic allodynia induced by NTG (Fig. 3D, clear circles vs. clear squares). Treatment with SNC80 (10 mg kg^−1^) blocked the development of both chronic and acute allodynia induced by NTG (Fig. 3C,D dark circles). In contrast to loxP controls, SNC80 failed to prevent the development of chronic allodynia induced by chronic intermittent NTG (Fig. 3C, filled squares). SNC80 also failed to relieve the acute allodynic effects of NTG on all test days (Fig. 3D, filled squares). These data suggest that δ receptors in forebrain GABAergic neurons play a critical role in the anti-migraine effects of δ agonist.

### Role of central δ receptors in OIH

**Figure 4:**
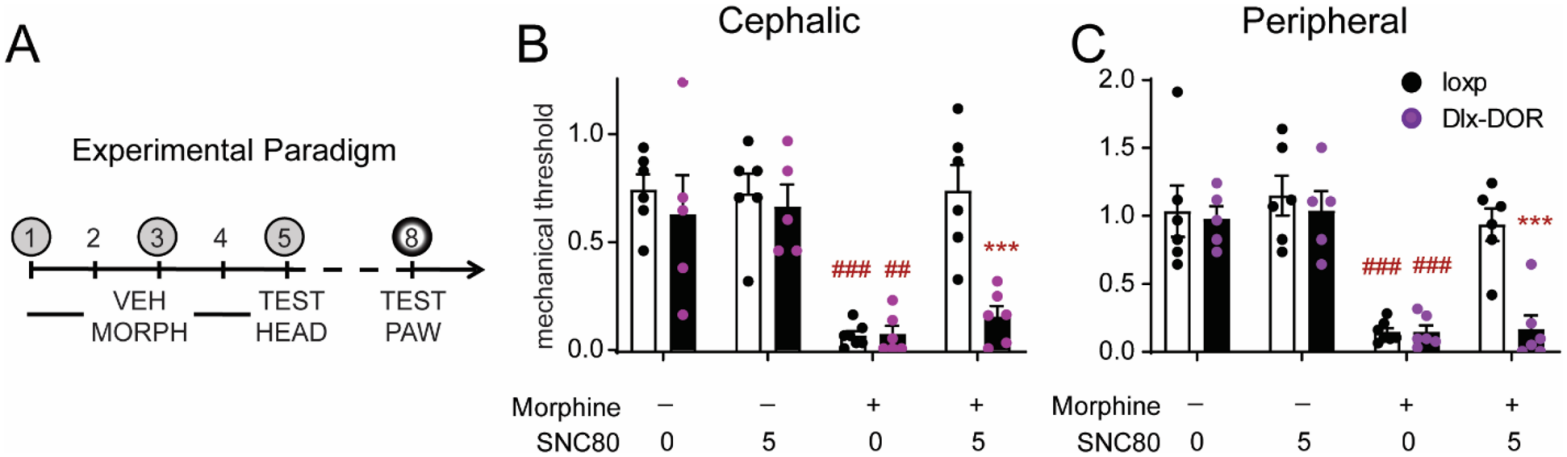
Role of central δ receptors in opioid-induced hyperalgesia. Mice were treated twice daily for 4 days with vehicle or morphine (20 mg kg^−1^ days 1–3 and 40 mg kg^−1^ day 4, SC) and tested for cephalic responses on days 1,3 and 5 (**A**, grey circles), and peripheral responses on day 8 (**A**, shadowed circle). Repeated morphine treatment produced periorbital (**B**) and hind paw (**C**) hyperalgesia in both loxP and Dlx-DOR mice. (**B**) In control mice, SNC80 reversed morphine-induced hyperalgesia in the periorbital region but had no effect in Dlx-DOR mice. Three-way ANOVA revealed significant effects of morphine dose (F(1,2) = 46.28, p < 0.001), SNC80 dose (F(1,2) = 9.28, p = 0.004), genotype (F(1,2) = 8.80 p = 0.005), and significant morphine dose X SNC80 dose (F(1,2) = 8.33, p = 0.006), SNC80 dose X genotype (F(1,2) = 4.45, p = 0.041), and morphine dose X SNC80 dose X genotype interactions (F(1,2) = 6.40, p = 0.016). (**C**) SNC80 inhibited opioid-induced hyperalgesia in the hind paw in loxP but not Dlx-DOR mice. Three-way ANOVA revealed significant effects of morphine dose (F(1,2) = 71.02, p < 0.001), SNC80 dose (F(1,2) = 8.76, p = 0.005), genotype (F(1,2) = 7.90, p = 0.008), and significant SNC80 dose X genotype (F(1,2) = 6.12, p = 0.018), and morphine dose X SNC80 dose X genotype interactions (F(1,2) = 4.59, p = 0.038). n = 6–10/group for all panels. ***p < 0.001 vs loxP control with same drug regimen. ^##^p < 0.01 vs. vehicle treated controls of the same genotype ^###^p < 0.001 vs. vehicle treated controls of the same genotype. n = 6/group; except loxP-MOR-VEH, cKO-MOR-VEH and cKO-MOR-SNC80 where n = 7/group.

Morphine was administered twice daily for 4 days (20 mg kg^−1^ on days 1–3, and 40 mg kg^−1^ day 4, SC), and the effect of SNC80 on cephalic responses and peripheral responses were tested on day 5 and day 8, respectively (Fig. 4A). Dlx-DOR conditional knockouts developed severe peripheral and cephalic allodynia to chronic morphine treatment similar to loxP controls (Supplementary Fig. 2, and Fig. 4B,C Morphine+, SNC80 0). On the day following the final morphine/vehicle administration, 5 mg kg^−1^ SNC80 inhibited morphine-induced periorbital and peripheral allodynia in loxP littermate controls (Fig. 4B,C). Morphine-treated animals that did not receive SNC80 continued to display robust periorbital and hindpaw allodynia. In Dlx-DOR knockout mice, SNC80 failed to inhibit morphine-induced periorbital allodynia (Fig. 4B), and the effect of SNC80 was also lost when measuring hind paw responses (Fig. 4C). Taken together, our data support a role for central δ receptors in the regulation of OIH by δ agonist.

### Role of central δ receptors in NTG-induced conditioned place aversion

**Figure 5:**
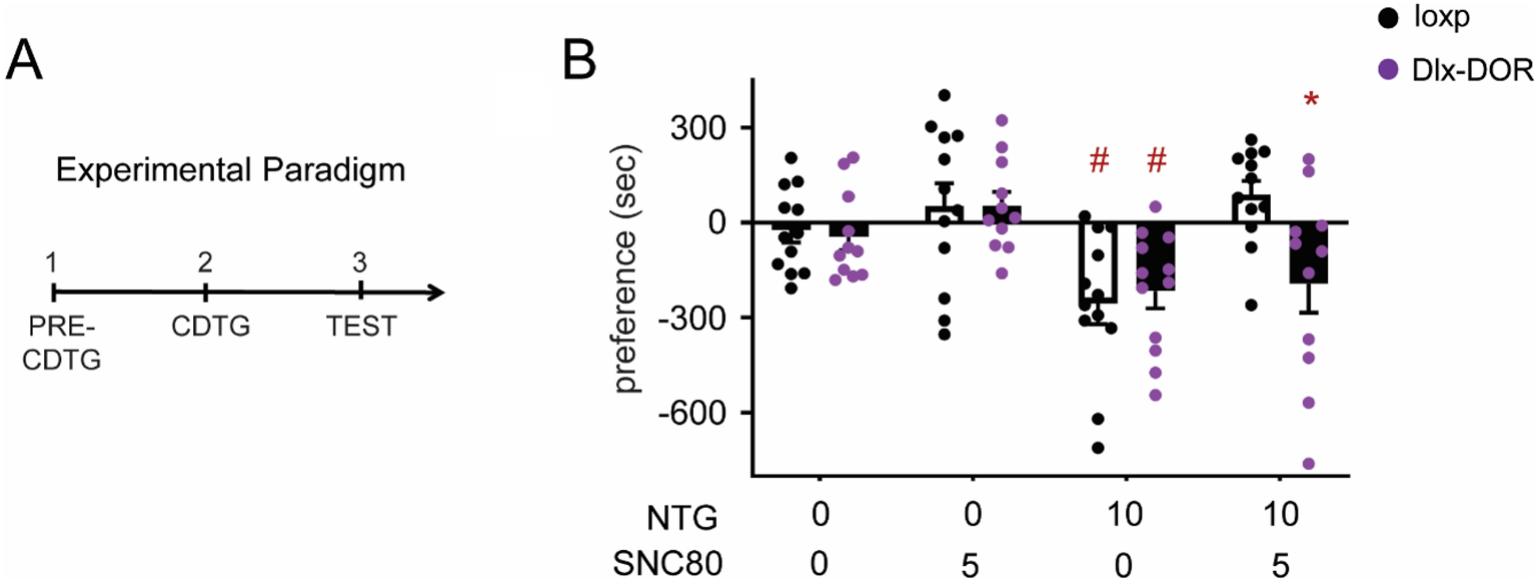
Role of central δ receptors in NTG-induced CPA. (**A**) Outline of experiment. (**B**) Conditioning with 10 mg kg^−1^ NTG produced significant CPA in all genotypes. Conditioning with SNC80 prevented the development of NTG-induced CPA in controls, but SNC80 was ineffective in Dlx-DOR mice. Three-way ANOVA revealed significant main effects of NTG dose (F(1,2) = 13.40, p < 0.001) and SNC80 dose (F(1,2) = 10.40, p < 0.002). There was also a trend for genotype X SNC80 dose effect (F(1,2) = 3.14, p = 0.08) and a significant NTG dose X SNC80 dose X genotype interaction (F(1,2) = 4.14, p = 0.045). n = 8–12/group. *p < 0.001 vs. loxP control with same drug regimen. ^#^p < 0.01 vs. vehicle treated controls of the same genotype. n = 12/group, except cKO-NTG-VEH where n = 13/group.

We have previously demonstrated that SNC80 blocks the development of NTG-induced conditioned place aversion^8^. To evaluate the relative contribution of forebrain GABAergic δ receptors, we examined the ability of SNC80 to inhibit NTG-induced conditioned place aversion in Dlx-DOR conditional knockout mice (Fig. 5A). We used a biased conditioning paradigm, where mice were conditioned to the preferred side^8^. We performed both state-independent and dependent test days. The data reported are from the state-independent tests (ie animals were tested drug-free). In loxP controls, conditioning with 10 mg kg^−1^ NTG produced a robust place aversion that was blocked by treatment with 5 mg kg^−1^ SNC80 (Fig. 5B, dark bars). NTG conditioning also produced significant place aversion in Dlx-DOR knockout mice. In contrast to loxP controls, SNC80 failed to block NTG-induced conditioned place aversion in Dlx-DOR knockout mice (Fig. 5B, hashed bars). In both genotypes, SNC80 did not produce a place preference in the absence of NTG. These data suggest that δ receptors in GABAergic forebrain neurons are critical for regulating the anti-aversive effects of δ agonist.

### Role of central δ receptors in cortical spreading depression

**Figure 6:**
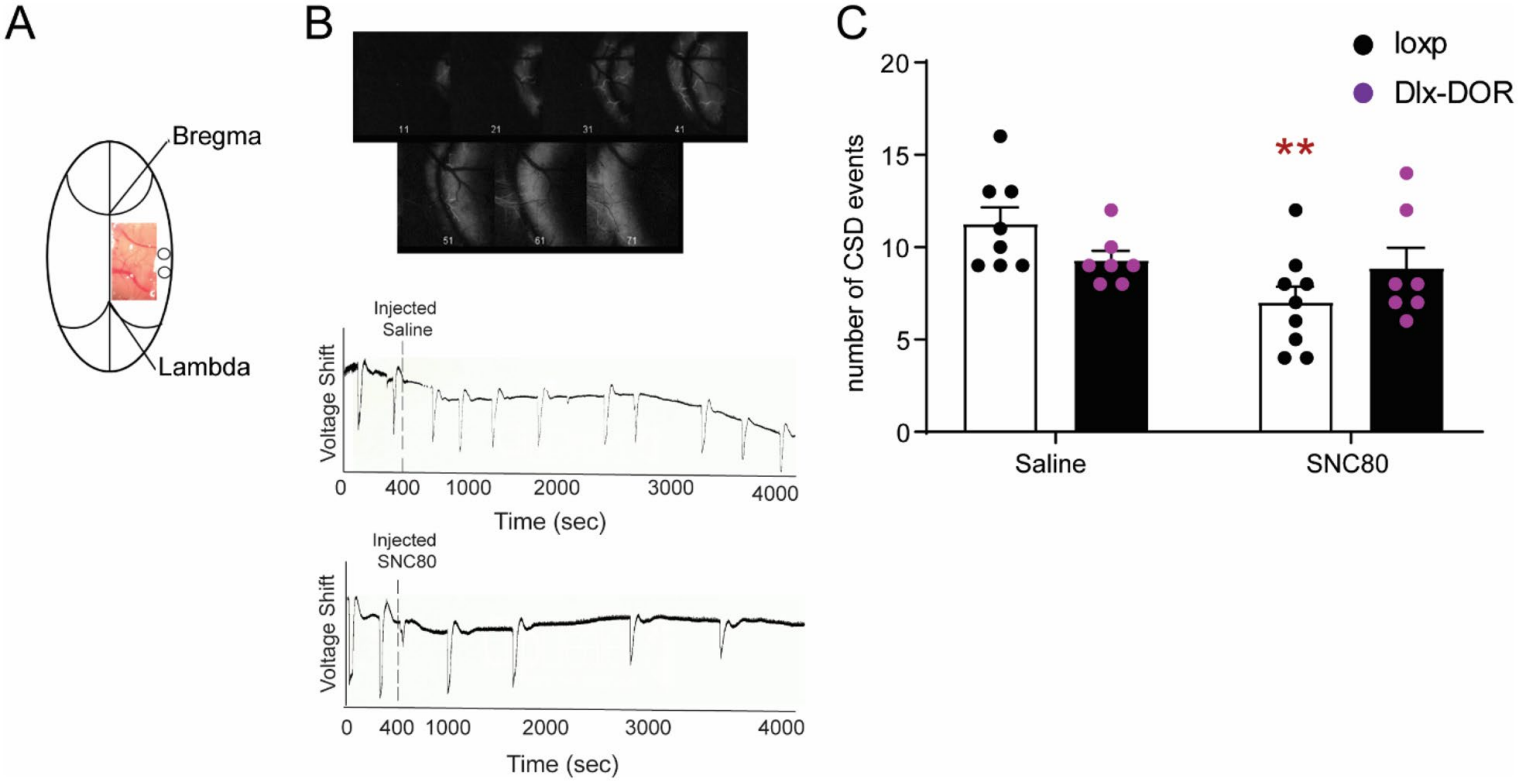
Role of central δ receptors in cortical spreading depression. (**A**) The location of the thinned skull and placement of the burr hole where KCl injection takes place and LFP recording is done. (**B**) To panel: Montage demonstrating the change in reflectance that can be seen in a typical CSD event. Lower panels: representative local field potential (LFP) recording that demonstrates the reflectance versus time that is typical for an hour of CSD recording following saline (upper panel) or SNC80 (lower panel) injection (IP). (**C**) SNC80 reduced the average number of CSD events in loxP controls, but failed to do so in Dlx-DOR animals. Two-way ANOVA revealed significant effects of SNC80 dose (F(1,27) = 6.92, p = 0.014), and a significant genotype X SNC80 dose interaction (F(1,27) = 4.61, p = 0.041). n = 7–8/group, **p < 0.01 compared to saline treated loxP group. n/group: loxP-VEH = 8, loxP-SNC80 = 9, cKO-VEH and cKO-SNC80 = 7/group.

To determine the effects of δ agonists on the cortical excitability underlying migraine, we measured the effects of SNC80 on CSD induced by continuous KCl application. This model has been previously shown to have predictive value for migraine preventive medications^22,23^. CSD events were visualized as waves of optical intrinsic signals originating from the KCl application site. In previous studies, these optical changes were shown to be associated with electrophysiological responses consistent with CSD^24–26^. To evaluate the role of GABAergic forebrain δ receptors in mechanisms of cortical excitability underlying migraine, the effects of SNC80 on KCl-evoked cortical spreading depression (CSD) were evaluated in Dlx-DOR mice (Fig. 6A). Systemic saline or SNC80 (10 mg kg^−1^) was administered 400 s after initial KCl application and CSD events were recorded for 1 h following injection (Fig. 6B). LoxP controls and Dlx-DOR mice showed a comparable number of CSD events in response to KCl (Fig. 6C, saline). SNC80 significantly decreased the number of CSD events in floxed littermate controls (Fig. 6C, dark bars). However, SNC80 lacked efficacy in the Dlx-DOR knockout mice (Fig. 6C, hashed bars). These results suggest that δ receptors in forebrain GABAergic neurons are necessary for the inhibitory effects of δ agonist on CSD.

## Discussion

In this study, we investigated the contribution of δ receptors expressed in the forebrain to the regulation of different headache-associated endpoints. Specifically, we observed a significant increase in δ receptor expression in the striatum, somatosensory cortex, and hippocampus in response to chronic migraine-associated pain. These findings led us to investigate the effect of knockout of δ receptors specifically in forebrain GABAergic neurons within multiple headache models. A key finding from this study was that δ receptors expressed in this receptor population played a crucial role in mediating the anti-migraine effects of δ agonist. We observed that in Dlx-DOR knockouts SNC80 failed to inhibit both peripheral and cephalic allodynia in models of chronic migraine-associated pain and OIH. In addition, the inhibitory effects of SNC80 were lost in the conditioned place aversion model and cortical spreading depression.

To help broaden our understanding of the role of δ receptors in migraine, we examined the effect of chronic migraine-associated pain on DOR-eGFP expression. We observed an increase in δ receptors following chronic NTG treatment in the TNC and TG. This finding was not surprising as these regions are involved with head pain processing, and studies in our lab are ongoing to understand the role of δ receptors at these sites. Interestingly, we also found that most forebrain regions examined showed elevated DOR-eGFP fluorescence in response to NTG including the hippocampus, NAc, and CPu. Multiple lines of evidence show that under naive conditions δ receptors have limited functionality, and that there are large receptor pools retained within intracellular structures. For example, δ receptors in TG and hippocampal neurons are localized to the golgi^27,28^, and δ receptors in DRG neurons are localized in large dense-core vesicles^29^. Inflammation and pain mediators are known to increase the transcription, translation, surface delivery, and functionality of δ receptors and improve the analgesic efficacy of δ agonists^30–34^. Our data would suggest that chronic intermittent NTG is also capable of increasing δ receptor expression in forebrain regions relevant to migraine-associated pain processing. One possibility is that this augmentation could serve as a potential protective mechanism in response to an altered pathological state. Increased δ receptor expression would allow for increased signaling by endogenous opioids. Transcriptomic and proteomic studies from our lab have also shown alterations in the expression of enkephalin in response to NTG^3,35^ suggesting adaptation of this signaling pathway. Upregulation of δ receptors within forebrain regions in response to NTG could modulate a number of physiological responses including pain processing, emotional regulation, memory, and motivation, all of which are affected in migraine.

Dlx5 and Dlx6 are transcription factors that are involved in the maturation of forebrain GABAergic neurons, and the Dlx5/6-Cre line shows high Cre expression in the dorsal and ventral striatum, hippocampus, and cortex^36,37^. Initial characterization of the Dlx-DOR line showed that these mice had a 60–84% loss of δ receptor within the dorsal (caudate putamen) and ventral (nucleus accumbens) striatum, a ~ 50% loss within the hippocampus, and a ~ 25% loss in the somatosensory cortex^20^. Dlx-DOR knockouts generally had behavioral responses similar to loxP littermate controls, and showed no change in locomotion, feeding, olfactory discrimination, or depressive-like behaviors^20^. However, Dlx-DOR mice did show loss of δ agonist-induced locomotion, decreased anxiety, decreased dopamine D1 receptor-induced hyperlocomotion, and altered motivation for food rewards^20^. In our study we also observed that deletion of δ receptors from forebrain GABAergic neurons did not affect baseline behavioral responses. Dlx-DOR mice showed naive mechanical responses to von Frey hair stimulation that was similar to loxP controls and developed a comparable allodynia to chronic NTG or morphine treatment. Furthermore, conditional knockouts also developed conditioned place aversion to NTG and had a similar number of CSD events to loxP mice. In contrast, all the SNC80-mediated effects were lost in these distinct behavioral paradigms. These data suggest that δ receptors in forebrain GABAergic neurons do not regulate basal responding to migraine and pain related stimuli but are critical for the anti-migraine effects of δ agonists. A caveat of our study is that like all germ line deletion experiments, conditional knockout of δ receptors could result in compensatory changes during development that could confound our results. In addition, we have also found that δ receptors in the TG and TNC are also upregulated following chronic NTG treatment. Future studies will determine how dependent δ agonist action is on synergistic responses between the forebrain, brainstem, and peripheral regions; and will use specific deletion and/or silencing of δ+ circuits in adulthood.

δ receptors are highly expressed in brain regions that regulate migraine, including trigeminal ganglia and trigeminal nucleus caudalis^38^. Our data indicates that activation of δ receptors in these regions is insufficient to evoke migraine symptom relief, as δ receptor expression in TNC and TG was maintained in the Dlx-DOR mice. One possibility is that the anti-migraine effects of δ agonists occurs at a number of different levels, which include an integration between peripheral, brain stem, and forebrain regions; and the disruption of this integrative response could explain why SNC80 is no longer effective in Dlx-DOR mice. For example, in the case of cephalic pain, trigeminal nociceptors from the TG relay pain signals through the TNC and thalamus to the somatosensory cortex. δ-mediated inhibition of cortical neurons could blunt responses to afferent pain signaling from this trigeminothalamic tract. There is also a descending corticotrigeminal projection which upon stimulation decreases trigeminal pain responding^39^, and δ agonists may work through this pathway. Further, projections from the TNC indirectly connect to the hippocampus via the amygdala, and this circuit may be involved in the altered emotional and cognitive states observed in migraine^40,41^. The effects of SNC80 on NTG-induced CPA may be mediated by δ receptor-expressing neurons in the hippocampus. SNC80-induced hyperpolarization of hippocampal δ receptor neurons could block pain inputs from the TNC and amygdala to inhibit the association of the NTG-induced negative affective state and the CPA chamber. Similarly, the NAc regulates the affective component of pain by encoding the value and salience of noxious stimuli (for review, see Ref.^42^). δ-mediated inhibition in the NAc could block transmission from salience-encoding neurons in the ventral VTA and prevent migraine-associated negative affect. It is also important to consider that δ receptors in forebrain regions may also affect neurotransmitter levels that in turn modulate migraine. The near complete loss of δ in the striatum could affect the dopamine fluctuations in these regions that have been observed during a migraine attack and allodynia^43^. Further, the loss of δ receptors from GABAergic neurons specifically implicates δ agonist regulation of inhibitory synapses as key to their anti-migraine effects. This study opens investigation of the potential role of δ receptor signaling on these different circuits and neurotransmitter systems and will be further investigated in the future.

Approximately 25–30% of migraine patients experience visual disturbances called aura as part of their migraine symptoms. CSD is considered to be the physiological correlate of aura^44^, and is a mechanistically distinct model of migraine relative to the NTG model. Forebrain δ receptors also appear to be important in regulating δ agonist effects on CSD. SNC80 has previously been shown to inhibit KCl-evoked CSD events^8^. Here we show that loss of δ receptors in GABAergic forebrain neurons significantly decreases the ability of SNC80 to inhibit CSD. Although δ receptors in Dlx5/6 neurons regulate CSD, it remains unclear whether the relevant receptor populations reside within the cortex, subcortical regions, or both. Enkephalins have been found to both elicit and inhibit CSD in rats^45,46^. CSD was specifically monitored in sensory cortex, as this region is more susceptible to CSD induction^23^. Overall, δ receptors in the cortex are not substantially reduced in Dlx-DOR mice and there is a ~ 27% decrease in δ receptor in the sensory cortex measured in our CSD studies^20^. Our data suggest that δ receptors within Dlx5/6 neurons act to specifically inhibit CSD, and this small decrease in receptor in the cortex may be in key GABAergic neurons that regulate the effect of SNC80. CSD has been shown to activate neurons within the trigeminovascular system^47–49^, and activation of cortical δ receptors may disrupt this process. Future studies will focus on the exact role of GABAergic δ receptors in CSD, and whether this effect is mediated through cortical or sub-cortical regions.

Overall, our data indicate that δ receptors within the forebrain are critical for the processing of migraine-associated endpoints by δ agonist. δ receptors are expressed broadly throughout the brain and periphery^14,50,51^, and δ agonists may work through multiple circuits to inhibit migraine-associated symptoms. We found that there was an abundant expression of δ receptors in regions that regulate head-specific pain. These δ receptors along with δ receptors in other brain regions may also be important for the effect of δ agonists. However, our data suggest that activation of δ receptors in forebrain regions are a key component of the circuits that regulate the action of δ agonists; and future studies will focus on dissecting these pathways further. These data provide novel insight on the role of forebrain δ receptors in migraine and support the development of brain penetrant δ agonists for headache disorders.

## Methods and materials

### Animals

Mice, aged 8–15 weeks, were housed in a temperature- and humidity-controlled animal colony on a 12 h light/dark cycle. Mice were group-housed with a maximum of 5 animals per cage in clear polypropylene cages with corn cob bedding and nestlets as enrichment. Food and water was available ad libitum. Both male and female mice were used in all experiments. Dlx-DOR^20^ mice were provided by B. Kieffer (McGill University). All animal experiments were performed according to the Association for Assessment and Accreditation of Laboratory Animal Care guidelines as administered by the University of Illinois at Chicago Animal Care Committees. Experiments also adhered to ARRIVE guidelines, and were approved by the UIC committee on Animal Care and Use. A total of 153 mice were used; 72 loxP, 71 Dlx-DOR, and 10 DOR-eGFP. For all behavioral tests the experimenter was blinded to the treatment group. Animals were randomly allocated to treatment groups for experiments shown in Figs. 1, 2, and 6. For Figs. 3, 4, and 5 mice were counterbalanced in groups after naïve responses were determined.

### Materials

All drugs were administered in a volume of 10 ml kg^−1^. SNC80 (Tocris Bioscience, Pittsburgh, PA) was dissolved in acidic 0.9% saline, pH 5.5. Nitroglycerin (NTG) was obtained as a 5 mg ml^−1^ stock solution (30% ethanol, 30% propylene glycol in water; American Regent, Shirley, NY) and diluted to 1 mg ml^−1^ in 0.9% saline both drugs were administered intraperitoneally (ip). Morphine (Hospira Inc, Lakeforest, IL) was dissolved in 0.9% saline and administered subcutaneously (sc).

#### Quantititave RT-PCR

Experiments were performed as described previously^52^. RNA was isolated from flash frozen brain punches using the RNeasy Mini kit from Quiagen. RNA samples were then reverse transcribed to single-stranded cDNA. cDNA transcription was used following the protocol from Superscript III (Life Technologies) and the TaqMan Gene Expression Assay System (Applied Biosystmes). Glyceraldehyde-3-phosphate dehydrogenase (GAPDH, Hs02758991_g1) (GAPDH-5′-CAATGTGTCCGTCGTGGATCT-3′ 5′-GTCCTCAGTGTAGCCCAAGATG-3′) was used as a housekeeping gene. The threshold cycle (CT) of each target product was determined and CT values between OPRD1 (5′-GCTCGTCATGTTTGGCATC-3′ 5′-AAGTACTTGGCGCTCTGGAA-3′) transcripts and housekeeping genes were calculated (ΔCT). The fold change (2-ΔΔCT) for each was calculated relative to the median ΔCT from the loxP control littermates.

### Sensory sensitivity testing

Mechanical hypersensitivity was evaluated as previously described^52^. In brief, mice were habituated to the testing apparatus for 1 h a day for 2 days prior to testing. For cephalic thresholds, mice were tested in 4 oz paper cups that were included in the testing apparatus during habituation. The threshold for response to punctate mechanical stimuli was assessed according to the up-and-down method. The plantar surface of the left hindpaw or the periorbital region caudal to the eyes near the midline was tested with a series of eight von Frey filaments (bending force ranging from 0.008 to 2 g). For hindpaw testing, a response was characterized as a lifting or shaking of the paw upon stimulation. For cephalic testing, a response was defined as a shaking or ducking of the head, or vigorous grooming of the periorbital region following stimulation.

#### NTG-induced hypersensitivity

For all experiments, NTG was administered on days 1, 3, 5, 7, and 9. For cephalic testing, thresholds were assessed on days 1, 5, and 9. On test days, basal thresholds were measured prior to injection of NTG. Mechanical thresholds were tested again 2 h post-NTG.

#### OIH

Mice received twice-daily injections of 20 mg kg^−1^ morphine or vehicle for three days. On day four, the dose was increased to 40 mg kg^−1^. Periorbital mechanical thresholds were assessed prior to the first morphine injection on days 1 and 3. On day 5, basal thresholds were measured prior to injection of SNC80 or vehicle. Mechanical thresholds were assessed again 45 min post-SNC80. On day 8, hindpaw hypersensitivity was measured according to the same protocol as day 5. Individual mice received the same drug regimen on days 5 and 8, and no drug was given days 6 and 7.

### Conditioned place aversion

The place conditioning apparatus was divided into two equal sized compartments separated by a plastic guillotine door. The two compartments differed in floor texture (metal grated floors with a square or diamond pattern) and wall design (horizontal stripes vs. polka dots). Live video tracking data was collected using a DMK 22AUC03 USB 2.0 monochrome industrial camera (The Imaging Source, Charlotte, NC), and analyzed using ANY-Maze software (Stoelting, Wood Dale, IL). The time spent in each compartment was recorded.

Conditioned place aversion experiments comprised four days of testing. On day one (preconditioning), mice were randomly placed in one compartment and allowed to freely explore the apparatus for 20 min. Based on the preconditioning results, test compounds (NTG/VEH and/or SNC80VEH) were paired with the preferred compartment (biased design). Day two (conditioning) consisted of two sessions. In the first session, all mice received two saline injections 1 h 15 min apart. Thirty min after the second saline injection, the mice were confined to the non-preferred chamber for 30 min. In the second session, mice were injected with 5 mg kg^−1^ SNC80 or vehicle 1 h 15 min after being administered 10 mg kg^−1^ NTG or vehicle. Thirty min after receiving SNC80, mice were confined to the preferred chamber for 30 min (1 h 45 min to 2 h 15 min post NTG). On day 3 (state-independent test), mice were allowed to freely explore both compartments in a drug-free state for 20 min. On day 4 (state-dependent test), mice were given their assigned drug treatments and allowed to freely explore both compartments for 20 min (1 h 50 min to 2 h 10 min post NTG). The preference score was calculated as the time spent in the conditioned chamber on the test day minus the time spent in this same chamber on the pre-conditioning day.

### Cortical spreading depression

The cortical spreading depression procedure used is based on previous work by Charles^25^ and Ayata^22,53^ which is an established model for screening potential migraine preventative compounds. Further this model was previously used in our own work^8^. Mice were grouped based on genotype, and then randomly subdivided into SNC80 or vehicle treatment (i.e., DLX-DOR-Veh, DLX-DOR-SNC80, LoxP-Veh, LoxP-SNC80). Prior to surgery, the weight of the mouse was recorded. For the cortical window, mice were anesthetized with isoflurane (induction 3–4%; maintenance 0.5–1%; in 67% N_2_ 33% O_2_) and placed into a stereotaxic frame on a homeothermic heating pad. Core temperature (37.0 ± 0.5 ℃) non- peripheral oxygen saturation (~ 99%), heart rate, and respiratory rate (80–120 bpm) were continuously monitored (PhysioSuite; Kent Scientific Instruments, Torrington, CT, USA). Mice were repeatedly tested throughout the procedure by tail and hind paw pinches to ensure sufficient anesthetic level.

CSD events were verified through both optical intrinsic imaging (OIS) and electrophysiologial recordings as previously described^8^. Briefly, following anesthesia, the skin from the skull was detached and a rectangular region of the skull approximately ~ 2.5 × 3.3 mm^2^ (~ 0.5 mm from sagittal, and ~ 1.4 from coronal and lambdoid sutures) to the right of the parietal bone was thinned until transparency was achieved using a dental drill (Fine Science Tools, Inc., Foster City, CA, USA). Subsequent addition of mineral oil was applied to the carved region to allow for greater visualization of the cortical surface parenchyma and vasculature to improve video recording. A green LED (530 nm) light was used to illuminate the skull throughout the CSD experiment (1-UP; LED Supply, Randolph, VT, USA). Cortical surface reflectance detected by OIS was collected with a lens (HR Plan Apo 0.5 × WD 136) through a 515LP emission filter on a Nikon SMZ 1500 stereomicroscope (Nikon Instruments, Melville, NY, USA). Images were acquired at 1–5 Hz using a high-sensitivity USB monochrome CCD (CCE-B013-U; Mightex, Pleasanton, CA, USA) with 4.65-micron square pixels and 1392 × 1040 pixel resolution.

Two burr holes were placed lateral to the midpoint of the previously thinned rectangular region. Burr holes were drilled deeper than the previously thinned skull region, such that dura was exposed but not broken. In the caudal burr hole a pulled glass pipette filled with saline was inserted that was attached to an electrode and an amplifier, which was used to record the local filed potential (LFP). A separate ground wire was placed underneath the skin caudal to the skull, which was used to ground the LFPs. Following electrode insertion background electrophysiological recording was monitored for at least an hour to let the mouse stabilize in the event of a CSD wave induced by the surgery or electrode placement. After the hour a second pulled glass pipette filled with 1 M KCl was placed into the rostral burr hole, avoiding contact with the brain or the surrounding skull. An initial drop of KCl stimulation was formed and then a subsequent even flow of KCl was maintained such that a pool of 1 M KCl was present throughout the duration of the experiment. If there was any excess KCl, tissue paper was used to carefully wipe it up. Approximately 400 s after the initial KCl drip, mice were injected with either vehicle or SNC80 (10 mg kg^−1^, IP) and the recording continued for 3600 s. Experiments were only included in final analysis if at least 2 CSD events occurred during the first 400 s of recording. After recording, video and LFP recordings were analyzed and used to count the number of CSD events that occurred within the hour of recording.

### Ex Vivo DOR-eGFP expression analysis

Sample preparation and image analysis was performed as previously described^54^. Briefly, DOR-eGFP knockin mice^55^ were anesthetized with 0.1 ml Somnasol (100 µl/mouse; 390 mg ml^−1^ pentobarbital sodium; Henry Schein, SKU#024352) and intracardially perfused with 10 ml 0.1 M phosphate buffer followed by 30 ml of 4% paraformaldehyde (PFA) in 0.1 MPB. Brains were post-fixed for 4 h at 4 °C in 4% PFA. The tissue was then cryoprotected at 4 °C in a 30% sucrose, 0.1 MPB solution until the tissue sank. Freely floating sections were cut at 30 μm in a cryostat and transferred to 0.1 MPB. Tissue was blocked in 5% NDST (5% normal donkey serum, 0.3% Triton X-100, 0.1 MPB) for 1 h at room temperature. Tissue was incubated with primary antibody (Rabbit anti-GFP, RRID AB_221569; A11122; Invitrogen), diluted 1:1000 in 1% NDST overnight at room temperature. Samples were washed 3 times for 10 min each with fresh 1% NDST and then incubated in secondary antibody (Alexa Fluor 488, Donkey anti-rabbit, RRID AB_2535792; Life technologies; A21206;) diluted 1:1000 in 1% NDST for 2 h at room temperature. Sections were washed 3 times for 10 min each with 0.1 MPB and then mounted on Superfrost glass slides and coversliped with Mowiol mounting medium.

Images for quantification were captured with an Evos FL Auto Cell Imaging System, using a 10× or 20× objective. eGFP expression was quantified by observers blinded to treatment groups. All eGFP-positive cells from all sections were analyzed. Quantification of cell mean fluorescence intensity was determined using ImageJ software. Density of cell fluorescence was calculated by dividing fluorescent intensity by surface area (pixel number). Nuclear fluorescence defined the background level and was subtracted from the cell fluorescence measures^56^. For brain regions in which fluorescence was too dense to evaluate at the level of individual neurons, density of fluorescence across the whole region was calculated.

### Data analysis

The data and statistical analysis comply with the recommendations on experimental design and analysis in pharmacology^57^. Three-way ANOVA tests were performed using SPSS Statistics 25 (IBM, Armonk, NY). All other data analysis was performed using GraphPad Prism version 7.00 (GraphPad, San Diego, CA). The level of significance (α) for all tests was set to 0.05. Post-hoc analysis was conducted using the Tukey’s post-hoc test to correct for multiple comparisons. Post-hoc analysis was only performed when F values achieved p < 0.05. All values in the text are reported as mean ± SEM. Experiments were designed to have an equal n/group. However, groups were sometimes slightly unequal to accommodate for using all animals in a litter. Outliers were defined using the Grubb’s test, and no outliers were identified using this analysis. Statistical analysis for each figure is reported in the figure legend. Specific p values for Figs. 1 and 2 where multiple t-tests with Holm-Sidak correction was performed are included in Supplementary Table 1.

## Supplementary information

Supplementary Information.
