# Supplementary material for: Forebrain delta opioid receptors regulate the response of delta agonist in models of migraine and opioid-induced hyperalgesia
Source: Sci Rep. 2020 Oct 19;10:17629. doi: 10.1038/s41598-020-74605-9 (PMC7573615; doi:10.1038/s41598-020-74605-9)
Supplement: Supplementary file 1 — Supplementary Information. [file 41598_2020_74605_MOESM1_ESM.docx]

**Supplementary Information**

**Forebrain delta opioid receptors regulate the response of delta agonist in models of migraine and opioid-induced hyperalgesia**

Isaac J. Dripps PhD^1^, Zachariah Bertels BS^1^, Laura S. Moye BS^1^, Alycia F. Tipton BS^1^, Kendra Siegersma BS^1^, Serapio M Baca^2,3^, Brigitte L. Kieffer^4^, Amynah A. Pradhan PhD^1,§^


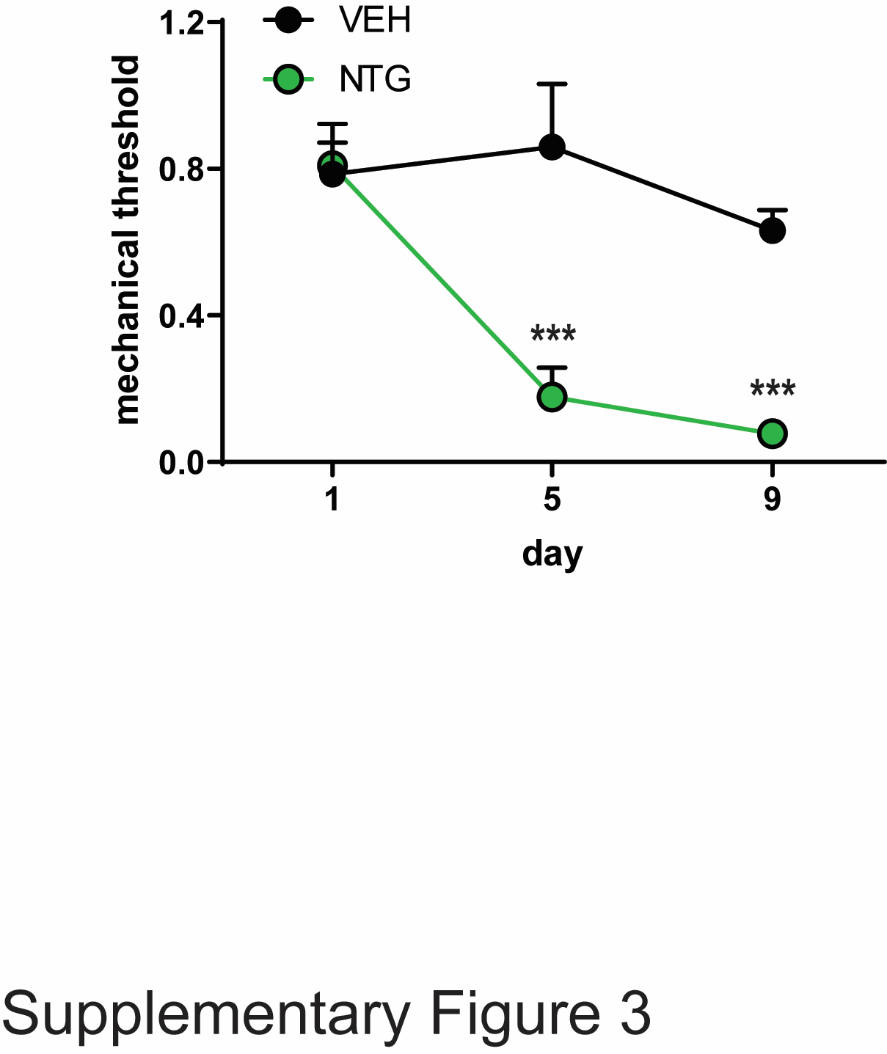


**Supplemental Figure 1. Chronic intermittent NTG produces significant cephalic allodynia in DOR-eGFP mice.** Mice were injected with vehicle of NTG (10 mg/kg IP) every other day for 9 days. They were tested on days 1, 5 and 9 for cephalic responses prior to injection of NTG/VEH (basal responses). Two-way RM ANOVA revealed significant effects of day (F(12,32) = 12.6, p<0.001), NTG (F(1,16) = 17.3, p<0.001), and day X NTG interaction (F(2,32) = 8.91, p<0.001). n=9 per group.

**
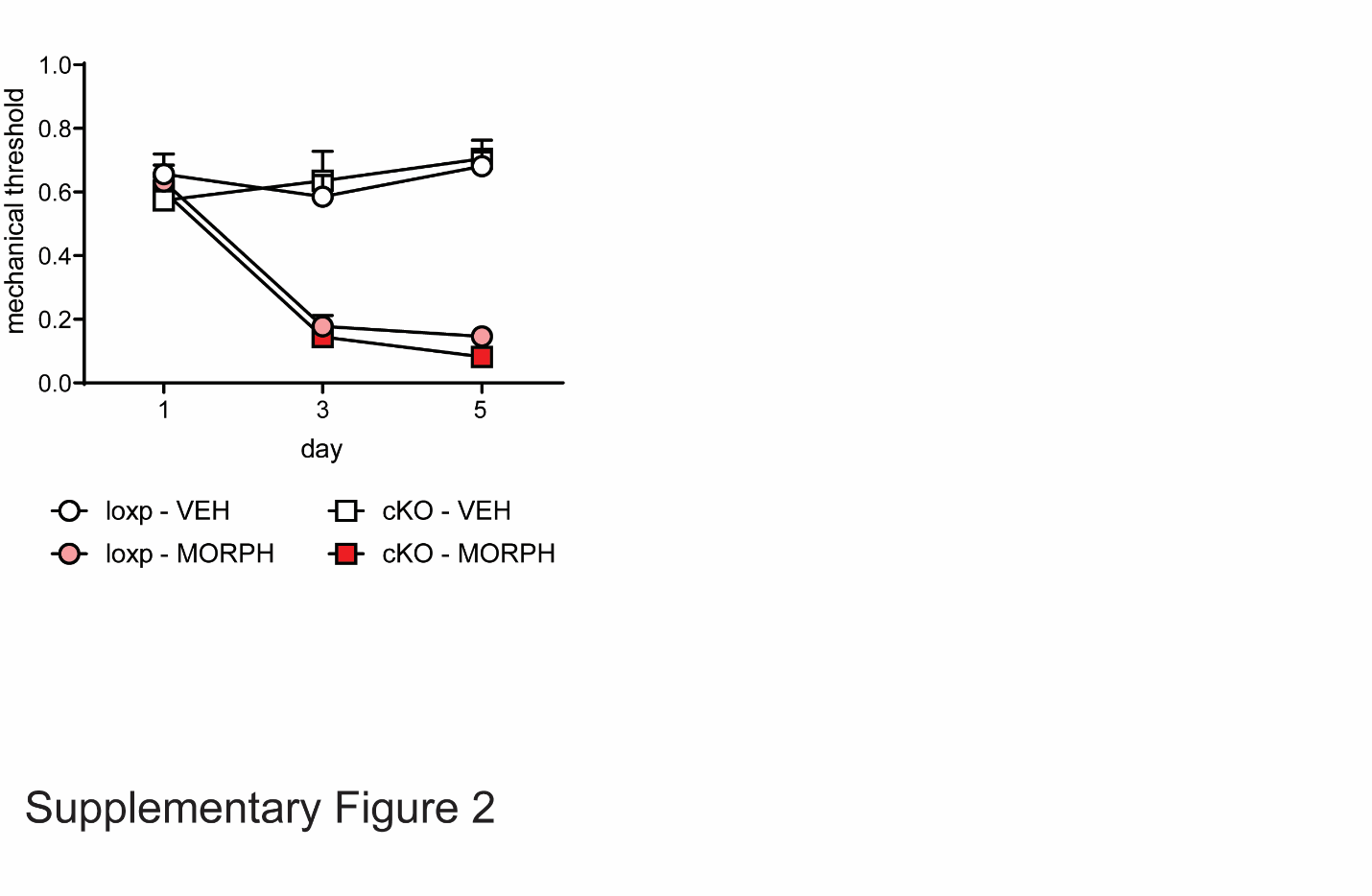
Supplemental Figure 2. Repeated morphine treatment produces mechanical hyperalgesia.** Mice received twice daily SC injections of 20 mg/kg morphine or saline on days 1-3. On day 4 mice received two SC injections of 40 mg/kg morphine or saline. (A) Both Dlx-DOR and loxP littermate controls developed severe cephalic hypersensitivity in response to repeated morphine administration. Three-way RM ANOVA revealed significant effects of day (F(2,2) = 19.32, p < 0.0001), morphine dose (F(1,2) = 105.8, p < 0.001), and a day X morphine dose interaction (F(2,2) = 27.38, p < 0.001). n = 10-13 per group.

**Supplementary Table 1**: p values for Figures 1 and 2 where multiple t-tests with Holm-Sidak correction was performed

| **Region Analyzed** | **p value** |
| --- | --- |
| **Figure 1B** |  |
| DG | 0.82010 |
| CA1 | 0.10065 |
| CA2 | 0.48250 |
| CA3 | 0.82010 |
| SC | 0.79333 |
|  |  |
| **Figure 1C** |  |
| DG | 0.06421 |
| CA1 | 0.00227 |
| CA2 | 0.00346 |
| CA3 | 0.02153 |
| SC | 0.06421 |
| NAcC | 0.00227 |
| NAcS | 0.05694 |
| CPu | 0.00006 |
|  |  |
| **Figure 2** |  |
| TG | 0.92172 |
| TNC | 0.58228 |
| hippo | 0.04922 |
| striatum | 0.00107 |
